# The spatiotemporal neural dynamics of action-related features underlying action recognition

**DOI:** 10.1162/IMAG.a.1019

**Published:** 2025-11-13

**Authors:** Marius Zimmermann, Angelika Lingnau

**Affiliations:** Chair of Cognitive Neuroscience, Institute of Psychology, University of Regensburg, Regensburg, Germany

**Keywords:** action recognition, LOTC, EEG, representational similarity analysis, fMRI-EEG fusion

## Abstract

The lateral occipitotemporal cortex (LOTC) has been suggested to host action representations that are thought to contribute to accessing meaning of observed actions. In line with this proposal, previous studies have shown spatially overlapping representations of various action-related features including objects, scenes, social properties, and kinematics, as well as abstract representations pertaining to action semantics. Less is known, however, about the way in which these features are integrated during action recognition. To address this question, we aimed to examine the temporal order in which different action-related features emerge in the EEG signal using EEG-based representational similarity analysis (RSA). Additionally, to investigate the spatiotemporal characteristics of these neural representations, we employed fMRI-EEG fusion. Static images spanning 27 everyday actions (e.g., riding a bike, washing dishes, brushing hair) were shown to participants (N = 24) in a delayed matching task, in a total of 648 trials (using different combinations of locations, actors, and viewpoints). Participants were asked to judge stimuli with respect to the type of action, location, and actor identity. Temporally specific neural representational dissimilarity matrices (RDMs) pertaining to neural representations of action categories were generated from 64-channel EEG recordings using time-resolved RSA. These were correlated with behavioral RDMs pertaining to semantic action (dis)similarities, revealing a time course of neural semantic action representations. Additionally, we considered RDMs capturing (dis)similarities between actions in terms of involved body postures, scenes, social aspects, and lower-level visual information. Results suggest a temporally ordered hierarchical buildup of neural representations related to visual, contextual, body-related, and semantic action information. fMRI-EEG fusion analysis further suggests that striate and extrastriate areas along the lateral visual pathway encode lower-level visual and body-related properties of actions, and that contextual and semantic information is integrated in the LOTC. These outcomes provide a spatiotemporal characterization of the neural processes enabling humans to recognize goal-directed actions.

## 1 Introduction

Action recognition, just like object recognition, requires us to extract invariant information from concrete properties. For example, we are able to recognize that someone is breaking into a house, irrespective of variations in the kinematics and the tools (e.g., a crowbar or a lockpicking set) or objects (e.g., a window or a door) involved. Decades of research in the field of action observation provided evidence that parietal, premotor, and temporal brain areas are involved in action observation, which together are referred to as the so-called action observation network (Bonini et al., 2022; Gallese & Goldman, 1998; Keysers & Perrett, 2004; Kilner, 2011; Orban et al., 2021; Rizzolatti & Craighero, 2004; for a recent review see Lingnau & Downing, 2024). More recently, it has been reported that particularly the lateral occipitotemporal cortex (LOTC), that is assumed to be part of the third, lateral visual pathway (Pitcher & Ungerleider, 2021; Weiner & Gomez, 2021; but see Ritchie et al., 2024), represents observed actions and action features at varying levels of abstraction.

Using multivoxel pattern analysis (MVPA) of fMRI data, Wurm and Lingnau (2015) revealed that the LOTC is sensitive to action goals such as opening and closing of containers irrespectively of the underlying kinematics (and thus, the specific action, such as screwing or lifting a cup), suggesting the presence of rather abstract, semantic representations. LOTC has further been shown to generalize across different types of objects (Wurm et al., 2016), across static and dynamic action stimuli (Hafri et al., 2017), and across visually and verbally presented actions (Wurm & Caramazza, 2019). Similarly, using representational similarity analysis (RSA) of fMRI data, Tucciarelli et al. (2019) and Kabulska et al. (2024) revealed that the similarity structure of a range of different actions, established behaviourally, is captured by the similarity structure of neural activation patterns across these actions in the LOTC. Moreover, both studies showed that semantic representations overlap with representations of both contextual features (e.g., object and scene properties) and body- and movement-related action properties. Remarkably, representations of the semantic similarity structure in the LOTC remained after accounting for similarities between actions with respect to body, scene, and object features (Tucciarelli et al., 2019). Complementary to this, distinct networks carrying information about body parts involved in an action as well as action targets have been identified in lateral and ventral occipitotemporal areas and the intraparietal sulcus (Tarhan & Konkle, 2020; Tucciarelli et al., 2019; Wurm et al., 2016). Even the representation of objects in LOTC has been shown to be driven by action-related properties (Cortinovis et al., 2025). Given this variety of overlapping and non-overlapping representations of action-related properties, it has been proposed that representations in the LOTC are organized along posterior-to-anterior gradients from concrete to abstract action features, as well as distinctions between dorsal and ventral LOTC in representations based on sociality and transitivity respectively (Lingnau & Downing, 2015; Wurm et al., 2017; Wurm & Caramazza, 2022; see also Papeo et al., 2019).

Unlike their spatial organization, the temporal order and causal dependencies of representations of action-related properties remain largely unexplored. Recent behavioral work suggested that shorter exposure durations are required to recognize simple actions (e.g., standing, sitting) than objects and scenes, whereas the recognition of goal-directed actions (e.g., ‘doing pottery’) required longer exposure durations (Reger et al., 2025). Regarding neural processes, observed pointing and grasping actions can be decoded from the MEG signal while generalizing across effector and reach direction within 200 ms (Tucciarelli et al., 2015). Likewise, object-related actions can be distinguished from non-object related actions within 250 ms from the time of stimulus presentation (Wamain et al., 2014), and viewpoint invariant representations can be decoded from brain activity within 200 ms (Isik et al., 2018). More recently, Dima et al. (2022) revealed a temporal hierarchy in the extraction of visual, action-related (i.e., action category, activity, transitivity, and effectors) and socio-affective features (i.e., valence, arousal, sociality, and number of agents) to EEG patterns during the observation of naturalistic videos of everyday actions.

In sum, whereas a growing number of studies suggest that the LOTC may represent different action-related properties in overlapping regions, and while several lines of research suggest a temporal gradient underlying these representations, the spatiotemporal gradient of these representations is currently unknown. This is because previous studies either used behavioral data, fMRI, or EEG. However, this information is crucial if we wish to understand when, where, and how different action-related properties are integrated for the recognition of actions (see also Lingnau & Downing, 2024; Ritchie et al., 2024). In the current study, we aim to fill this gap by investigating the temporal and spatiotemporal dynamics of action-related properties using EEG-based RSA and EEG-fMRI fusion analyses (Cichy & Oliva, 2020). According to a strict hierarchical (feedforward) model, we expected that (1) the representation of action-related properties unfolds in an orderly fashion, starting with basic visual features of the visual scene (or stimuli), followed by body-, scene-, and object-properties, and finally action semantics. In addition, we hypothesized that (2) temporally specific neural activations (based on EEG) correspond to spatially specific neural activation (based on fMRI data) in a spatiotemporal order along the lateral visual pathway (Pitcher & Ungerleider, 2021; Weiner & Gomez, 2021; but see Ritchie et al., 2024), ranging from lower visual areas (for basic visual features) to extrastriate areas (for object-, scene-, and body-related features), to the LOTC (for action semantics). By contrast, according to a parallel (feedforward) model, the representation of different action-related properties is assumed to emerge in parallel, in overlapping regions along the lateral visual pathway. Finally, in contrast to the predictions of serial or parallel feedforward models, dynamic changes of representations of action-related properties both at shorter and longer latencies would suggest the involvement of recurrent processing (Lamme & Roelfsema, 2000).

## 2 Methods

### 2.1 EEG-based representational similarity analysis

#### 2.1.1 Participants

A total of 24 female and male participants (19–34 years, mean ± sd: 23.9 ± 4.5; sex or gender information was not collected given that we did not have any hypothesis pertaining to sex or gender effects) were recruited using an online recruitment platform for research participants at the University of Regensburg. All participants were right-handed without severe neuropsychological disorders (self-report) with normal or corrected-to-normal vision. All participants received information regarding the experimental procedures and gave their written informed consent to participate in the study. Participants were compensated with 10€ per hour or course credit. The study was approved by the local ethics committee at the University of Regensburg (21-2657-101).

#### 2.1.2 Stimuli

The stimulus set contained 324 static images spanning 27 basic-level everyday actions (e.g., riding a bicycle, washing dishes, brushing hair). The stimulus set was a subset of the stimuli used by Tucciarelli et al. (2019), as provided on OSF (https://osf.io/cvrb2/). One basic-level action (swimming) was removed from the original stimulus set, since the depicted action was performed by different actors. There were 12 different exemplars for each action, using different combinations of actors (2), locations (2 exemplars each; e.g., two different kitchens), and viewpoints (3), for a total of 324 different images.

#### 2.1.3 Task

**Fig. 1.**
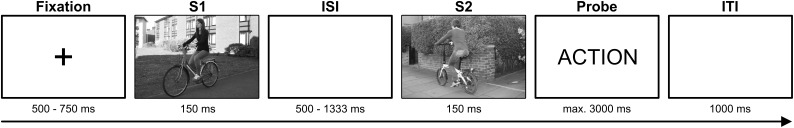
Experimental task. Schematic overview of a single trial. Following a fixation cross, two images (S1, S2) depicting everyday actions were shown with a variable inter-stimulus interval (ISI). At the end of each trial, a probe was presented, indicating the task: to judge whether S1 and S2 (1) correspond to the same basic-level action, (2) are performed by the same actor, or (3) take place at the same location. In this example (probe: action), the correct answer is [yes] (both 7 pictures show a person riding a bicycle). If the probe were location or actor, the correct answer would be [no] in both cases, as the actions were performed by different actors, and at different locations.

A trial consisted of five phases (Fig. 1). At the beginning of each trial, a fixation cross was presented for a random interval (500–750 ms), followed by the first stimulus (S1) shown for 150 ms. Next, an empty screen was presented for a variable inter-stimulus interval (ISI) of 500–1333 ms [500, 667, 833, 1000, 1167, or 1333 ms], followed by the presentation of the second stimulus (S2) for 150 ms. The second stimulus was followed by a probe question, indicating which feature (action, location, or actor) participants were asked to match between the first and second stimulus in the current trial. The probe was shown until a response was given, but no longer than 3000 ms. Participants responded by button press on a keyboard with their left (*no* responses) or right (*yes* responses) index finger. Each trial was followed by a fixed inter-trial-interval (ITI) of 1000 ms.

All stimuli were used twice as S1, resulting in a total of 648 trials (27 actions x 12 exemplars x 2 repetitions). Probe questions were evenly distributed over trials (216 questions per feature). Trials were structured such that half of the trials, with respect to the probe question, showed matching stimuli, and half non-matching stimuli. S2 in each trial was chosen according to probe question and expected answer (yes, no), and selected randomly from the remaining possible stimuli (with an exception for *location* probes, where S2 was always selected from the same action as S1). Note that brain activation was only analyzed during presentation of the first stimulus and the ISI. Hence, the second stimulus and the probe were not directly affecting EEG measures.

The experimental task was presented using ASF (A Simple Framework; Schwarzbach, 2011) for MATLAB (R2021a; The MathWorks Inc., Natick, MA, USA)-based Psychtoolbox (3.0.19; Brainard, 1997). Stimuli were presented on a 120 Hz VIEWPixx /EEG monitor (VPixx Technologies Inc., Saint-Bruno, QC, Canada) in a shielded EEG room. A 60 second rest break was included after each set of 58 trials. Prior to the experiment, participants completed a practice session with 20 trials. The experiment lasted approximately 40 minutes.

#### 2.1.4 Behavioral data processing and analysis

Behavioral responses were analyzed in MATLAB (R2021a). Per condition (probe type x ISI) trials were removed that exceeded two interquartile ranges (IQR) from the median (2.60% removed; range: 0–4.95%). The remaining responses were evaluated for accuracy, and reaction times were computed for correct trials, for each combination of probe type (action, location, actor) and ISI (500–1333 ms). Accuracies and response times were analyzed by two 2-way repeated-measures ANOVAs with factors *probe type* and *ISI*. One participant was excluded from behavioral data analysis due to technical issues, such that responses were not collected reliably.

#### 2.1.5 EEG data acquisition and preprocessing

EEG data were recorded using a BrainAmp EEG system (BrainProducts; Gilching, Germany) using 63 gel-based passive Ag/AgCl electrodes (BrainCap) and a sampling frequency of 500 Hz. The data were referenced to Cz during acquisition. EEG data and responses were stored for offline analyses in FieldTrip (v20231025; Oostenveld et al., 2011) running in MATLAB (R2021a).

Data were bandpass filtered using a two-pass 0.1 Hz high-pass filter and 40 Hz low-pass filter (using a 5th-order Butterworth filter). Noisy channels were removed based on visual selection (on average, 0.8 channels were removed; range 0–7). In addition, to remove artifacts from the EEG signal, independent component analysis was performed on each dataset using the EEGLAB function *runica* (Bell & Sejnowski, 1995), while excluding break periods. Components were automatically labeled using IClabel (Pion-Tonachini et al., 2019). Components which were labeled as eye movement artifacts (prob > 50%), muscle artifacts (prob > 50%), heart artifacts (prob > 50%), or noise (prob > 50%) were regressed out from the data. On average, 10.5 components were removed per participant (range: 3–24). The resulting data was epoched in trials from -500 to 3000 ms relative to the onset of stimulus S1 in each trial. Trials with noisy signals were detected and removed using visual rejection using the ft_rejectvisual function. An average of 9.75 trials were removed per participant (range: 1–22). Rejected channels were interpolated using the average of neighboring channels, and channels were re-referenced to the global average of all channels.

#### 2.1.6 EEG data analysis

##### 2.1.6.1 Generation of neural RDMs

**Fig. 2.**
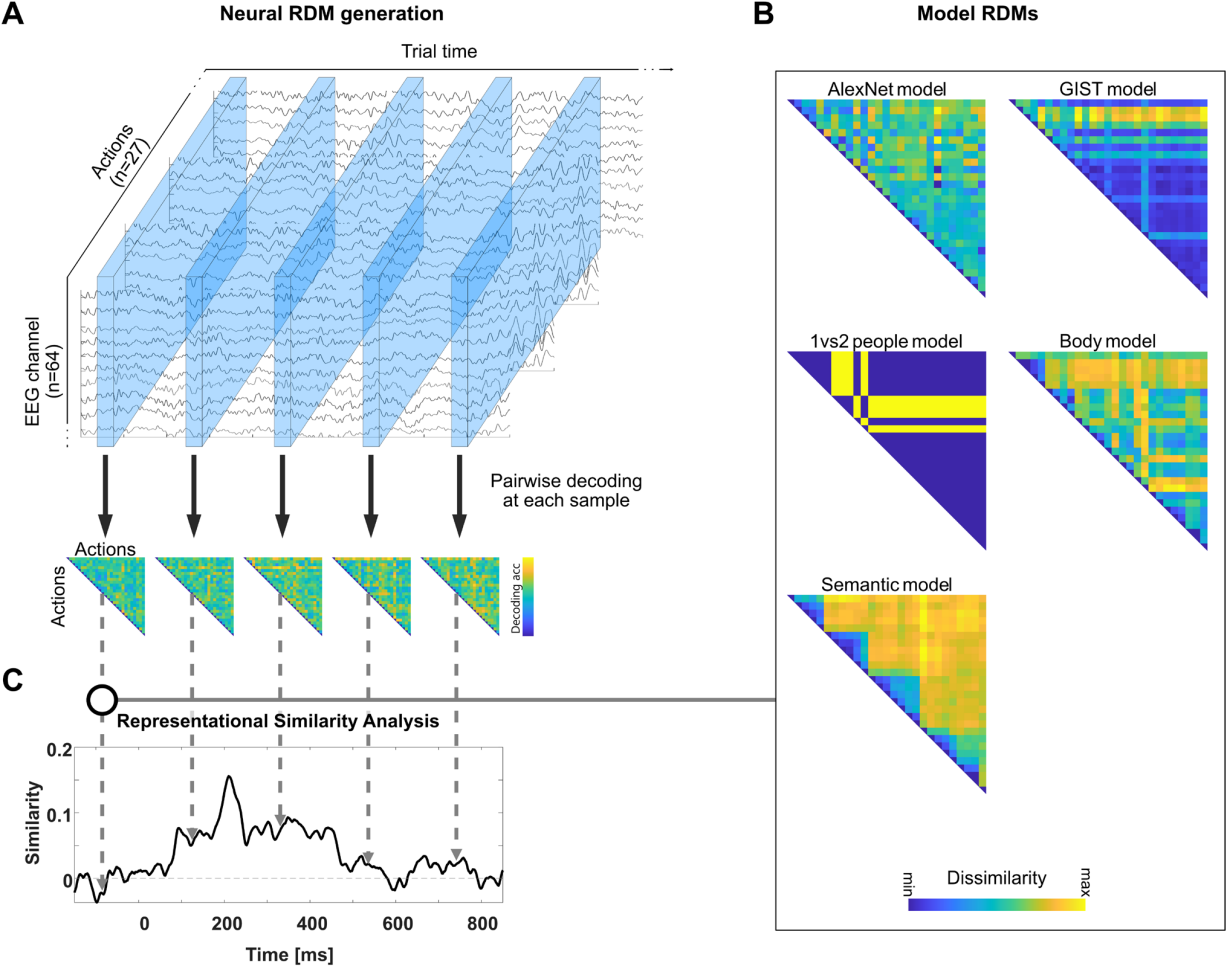
Illustration of procedures related to EEG-based Representational Similiarity Analysis. (A) First, neural RDMs were generated for each sample of the EEG data using pairwise decoding between actions. When a classifier can distinguish between two actions based on neural patterns at a given sample, this indicates high dissimilarity. When a classifier is not able to distinguish between two actions, dissimilarity is low. (B) Next, model RDMs were generated using the 2^nd^ layer of the AlexNet CNN, output of the GIST computational model of the recognition of global scene properties, and data resulting from a behavioral multiarrangement task (Tucciarelli et al., 2019), providing a set of model RDMs for low-level visual features (AlexNet model), scene information (GIST model), the number of people (1 vs 2 people model; based on the number of people depicted in the image), body parts used to perform an action (Body model), and semantic similarity (Semantic model). Note that additional models were generated initially, but excluded due to strong correlations (see Section 2.1.6). (C) Finally, temporally specific neural RDMs (A) and model RDMs (B) were correlated using two approaches: standard RSA based on Spearman rank correlations, and multiple regression RSA, 11 in which the contribution of each model RDM to the neural RDM is evaluated while accounting for all other models. Both approaches provide model specific time courses of representational similarity between model RDMs and neural RDMs.

For each participant, temporally specific neural representational dissimilarity matrices (*neural RDMs*) pertaining to neural representations of basic-level actions were generated from whole-brain sensor-level ERP data using time-resolved multivariate pattern analysis ((MVPA; Haxby et al., 2001); see Fig. 2A for an illustration of procedures) as implemented in CosmoMVPA (Oosterhof et al., 2016). Specifically, neural RDMs were constructed from pairwise decoding accuracies for each pair out of the 27 actions (i.e., collapsing over exemplars and repetitions) at each sample, between -500 and 3000 ms relative to the onset of S1. Decoding was achieved using a support vector machine classifier as implemented in the LibSVM library (Chang & Lin, 2011) in a take-one-fold out cross-validation approach over six chunks using balanced partitions, averaging data over 5 samples (10 ms) at a time. Data from all EEG channels were considered features. Pairwise decoding accuracies were assigned to the 3^rd^ of the 5 samples used for decoding, and stored in neural RDMs for each time point. In these RDMs, high decoding accuracies indicate patterns that are more different from each other, while low decoding accuracies reflect neural patterns that are more similar to each other.

##### 2.1.6.2 Generation of model RDMs

To characterize the temporal unfolding of action-related features underlying the ability to recognize actions, we used several different model RDMs (see Fig. 2B for an illustration of procedures). To capture low-level visual information, we created a model RDM based on the second layer of AlexNet (Krizhevsky et al., 2017), which has been proposed to qualitatively correspond to cortical V2 data (Laskar et al., 2020) (*AlexNet model*). To capture scene information available in the action images, we used the GIST model (Oliva & Torralba, 2001; *GIST model*). These RDMs were generated by pairwise comparison of model outputs for each action stimulus, averaged over different exemplars of each action. To capture the number of actors involved in the action, we used an additional binary model (*1 vs 2 People model*), created by assigning binary values to each action based on the number of actors (1 or 2) in the scene. To capture the similarity of actions with respect to body parts (i.e., which body parts are used for an action; *Body model*), context (i.e., where the action takes place; *Context model*), movements (*Movement model*), objects (*Object model*), and semantic similarity (*Semantic model*), we used data by Tucciarelli et al. (2019) resulting from a multi-arrangement task (Kriegeskorte & Mur, 2012), where a group of N = 20 participants was instructed to arrange the same images that were used in the current study (see Section 2.1.2 for details) on a circular arena according to their perceived similarity, carried out separately for each of these action-related properties. All models were transformed into dissimilarity matrices and normalized (mean-centered and standardized).

Several model RDMs showed strong correlations (e.g., semantic x context: r = .83, semantic x object: r = .74, body x movement: r = .74; see Supplementary Table S1 for a full list), and accordingly, high Variance Inflation Factors (VIF; range: 1.08–4.20). Therefore, we decided to reduce the set of model RDMs to the *AlexNet model*, *GIST model*, *1 vs 2 people model*, *Body model*, and *Semantic model*, such that a VIF below 2 was reached (final VIFs: 1.06–1.41). The final set of model RDMs is shown in Figure 2B and their pairwise correlations in Table 1.

**Table 1.** Correlations between model RDMs.

	GIST model	1 vs 2 people model	Body model	Semantic model
AlexNet model	.36	.14	.16	.21
GIST model		-.05	.34	.19
1 vs 2 people model			-.01	.15
Body model				.47

Pairwise pearson correlation between normalized model RDMs of included models.

##### 2.1.6.3 Time-resolved representational similarity analysis: Standard RSA

Standard RSA was performed by correlating neural RDMs with normalized model RDMs using Spearman rank correlations, sample by sample (Fig. 2C). This process was repeated for each model RDM, separately for each participant. Next, correlation values were transformed using the Fisher z-transform. The resulting correlation time series were analyzed for deviation from zero using one-sample suprathreshold cluster tests corrected for multiple comparisons across time using non-parametric randomization tests (Dima et al., 2022; Nichols & Holmes, 2002). First, significant samples were identified using one-tailed t-tests with an alpha level threshold of .05. Next, cluster sums were tested for significance using a Monte Carlo approach with 10,000 randomizations, using an alpha-level threshold of .05, over the time window between stimulus onset and 800 ms post-stimulus onset, using 10 ms sliding windows (ft_timelockstatistics; maximum cluster sum statistics). The noise ceiling was calculated by averaging the correlations of individual EEG data (i.e., individual time-resolved RDMs) with the average time-resolved RDMs of the remaining participants in a leave-one-out approach (lower bound), and with the average time-resolved RDMs of all participants (upper bound; Nili et al., 2014).

##### 2.1.6.4 Time-resolved representational similarity analysis: Multiple regression RSA

Multiple regression RSA was conducted using the same data (neural and model RDMs) and settings as standard RSA. However, all models were considered simultaneously when predicting neural RDMs at each sample using a general linear model, including the five model RDMs (*AlexNet model*, *GIST model*, *1 vs 2 people model, Body model*, and *Semantic model)* and a constant term. Regression parameters for each model were entered into a randomization test as described for standard RSA.

Following Cichy et al. (2014), 95% confidence intervals for mean onset latencies of the first significant cluster were determined by bootstrapping the participant sample (n = 24). Specifically, we created 1000 bootstrapped samples by sampling from all participants with replacement. For each bootstrap sample, we repeated the exact multiple regression RSA as in the original sample, for each model. The resulting bootstrap estimates of onset latencies were used to determine the 95% confidence intervals for onset latencies for each model.

### 2.2 fMRI-EEG fusion

#### 2.2.1 fMRI data

We performed an fMRI-EEG fusion analysis (Cichy & Oliva, 2020), using the EEG data collected in the current study (see Section 2.1), and fMRI data obtained from Tucciarelli et al (2019; available via OSF: https://osf.io/cvrb2/). In brief, Tucciarelli et al. (2019) presented participants (N = 20) with the same stimuli (except for the action ‘swimming’; see Section 2.1.2) in a one back task, requiring a button press during rare catch trials in which the previous trial showed the same action (but not the same exemplar) as the current trial using an event-related fMRI design (for a detailed description of the task and data preprocessing, see Tucciarelli et al., 2019). Beta weights were obtained for each of the 27 actions in MNI space. These beta maps were used as input for the current fMRI-EEG fusion analysis.

#### 2.2.2 Generation of neural (fMRI) RDMs

**Fig. 3.**
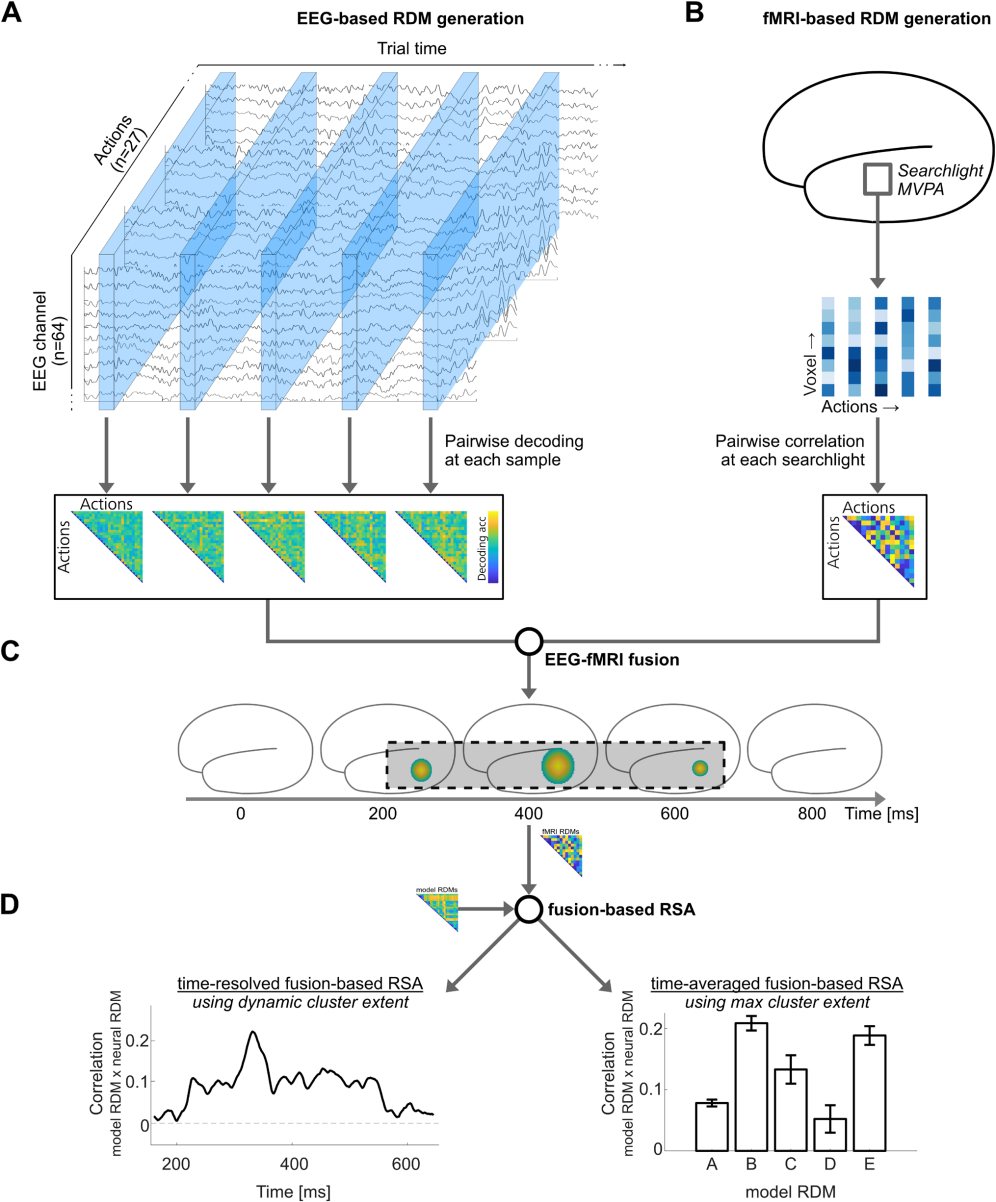
Illustration of procedures related to fMRI-EEG fusion and fusion based RSA. (A, B) First, EEG-based and fMRI-based neural RDMs are obtained for each time point (EEG-based RDMs; A) and each central voxel within a sphere (fMRI-based RDMs; B). For EEG-based RDM generation, see Figure 2. fMRI-based neural RDMs are obtained for each central voxel using a searchlight approach. For each voxel within a sphere, beta values are extracted for each action in each participant from a dataset obtained by Tucciarelli et al. (2019), using a similar task design 15 (see Section 2.2.1). fMRI-based RDMs are generated by pairwise comparison (using Spearman correlations) of beta values between action pairs, within each searchlight (see Section 2.2.2 for details). (C) Next, EEG-fMRI-fusion is performed by correlating EEG-based RDMs corresponding to each time point and fMRI-based RDMs corresponding to each central voxel within a sphere, generating spatiotemporal similarity maps between EEG-based and fMRI-based neural RDMs. Maps are thresholded to obtain spatiotemporal fusion-based clusters (dashed box in C). (D) Fusion-based RSA is performed by correlating fMRI-based RDMs obtained from voxels within the fusion-based clusters and model RDMs corresponding to action features (see Fig. 2 for details and generation; Section 2.2.3 for details) in two approaches: time-resolved and timeaveraged. For time-resolved fusion-based RSA (D, left panel), beta values from all participants were extracted at each time point from the fusion-based cluster, and used to generate temporally specific cluster-RDMs from all voxels within the cluster extent at a given time. These cluster-RDMs were compared for similarity to model RDMs (using Spearman correlations), providing similarity time courses between each model RDM and fusion-based cluster, spanning the time course of the cluster. For time-averaged fusion-based RSA (D, right panel), beta-values were extracted from all voxels that fell within the spatiotemporal fusion-based cluster at any point in time. These were used to generate time-averaged cluster-RDMs and compared for similarity to model RDMs (using Spearman correlations), providing time-averaged similarity measures between each model RDM and fusion-based cluster (see Section 2.2.4 for details).

Neural fMRI-based RDMs (from here on referred to as *fMRI-RDMs* to avoid confusion with neural EEG-based RDMs) were generated for each participant (N = 20) that took part in the fMRI study by Tucciarelli et al. (2019) using a whole-brain searchlight approach (see Fig. 3 for an illustration of fMRI-EEG fusion procedures). Specifically, for each voxel (2 mm isotropic voxel size, normalized to the MNI template, unsmoothed data), beta values of all voxels within a 6 mm sphere were extracted from beta maps corresponding to all 27 actions (Fig. 3B). Within each sphere, pairwise correlations between beta values corresponding to each action were computed using Spearman correlations, transformed into dissimilarity scores (1-r), and assigned to the central voxel of the sphere. These voxelwise dissimilarity scores were averaged across participants, and resampled to 4 mm isotropic voxel size (due to memory constraints in the subsequent cluster-based randomization analysis). The resulting voxelwise fMRI-RDMs, thus, capture dissimilarities in multi-voxel neural responses between all action pairs.

#### 2.2.3 fMRI-EEG fusion analysis

For the EEG-fMRI fusion analysis (Fig. 3C), the EEG-RDMs for each time point (between -100 and 700 ms relative to the onset of S1 in the EEG task, see Section 2.1) were correlated with the group fMRI-RDMs for each voxel (see Section 2.2.2) using Spearman rank correlations (n.b., the time window was reduced to -100 to 700 ms based on the outcomes of the EEG-RSA analysis, in order to reduce memory requirements). This resulted in a time course of correlation values between each of the voxel specific fMRI-RDMs and the time-resolved EEG-RDMs for each participant in the EEG experiment. Correlation values were transformed using the Fisher z-transform. Resulting correlation time series were analyzed for deviation from zero using one-sample suprathreshold cluster tests corrected for multiple comparisons across time using non-parametric randomization tests (Nichols & Holmes, 2002). First, significant voxel x sample combinations were identified using one-sample t-tests with an alpha-level threshold of .05 to form clusters over neighboring samples and voxels. Next, the cluster sum scores of these clusters were tested for significance using a Monte Carlo approach with 1,000 randomizations (limited by memory constraints), using an alpha-level threshold of .05, over the time window between stimulus onset and 700 ms post-stimulus onset of S1, using 10 ms sliding windows (ft_timelockstatistics; *maxsum* statistics).

#### 2.2.4 fMRI-EEG fusion-based RSA

Having identified clusters of voxels showing a significant similarity between fMRI- and EEG-based neural RDMs, we performed standard RSA using fMRI data from each of those clusters and the model RDMs described in Section 2.1.6. This analysis was performed in two different ways, namely, averaged across time (using each cluster in its entirety) and time-resolved (using the sample-by-sample cluster dimension; see Fig. 3D, for an illustration of fusion based RSA).

For the time-averaged fMRI-EEG fusion-based RSA, binary masks were generated based on each spatiotemporal cluster identified in the fusion analysis, each including all voxels that were part of the cluster at any point in time. Next, beta values within these clusters were extracted from all participants (N = 20) that took part in the fMRI experiment by Tucciarelli et al. (2019; see Section 2.2.1), and cluster-specific fMRI-RDMs were generated using 1-r pairwise Spearman rank correlations. These fMRI-RDMs were correlated, using Spearman correlations, to each of the model RDMs, transformed using the Fisher z-transform, and tested for deviation from zero using t-tests. P-values were corrected for multiple comparison using FDR correction over models and clusters.

For the time-resolved fMRI-EEG fusion-based RSA, masks were generated based on the spatial cluster extent at each sample that was included in the cluster. Next, beta values corresponding to the cluster at each sample within the cluster were extracted from all 20 fMRI participants (see Section 2.2.1), and cluster x sample specific fMRI-RDMs were generated using 1-r pairwise Spearman rank correlations, resulting in fMRI-RDM time courses for each cluster. These fMRI-RDM time courses were correlated, using Spearman correlations, to each of the model RDMs, transformed using the Fisher z-transform, and tested for deviation from zero using one-sample suprathreshold cluster tests corrected for multiple comparisons across time using non-parametric randomization tests (Nichols & Holmes, 2002) (ft_timelockstatistics; *Monte-carlo* approach, *maxsum* statistics, 10,000 randomizations, cluster-forming alpha level .05, FDR-corrected for multiple comparisons over models and clusters).

## 3 Results

### 3.1 Behavioral performance

**Fig. 4.**
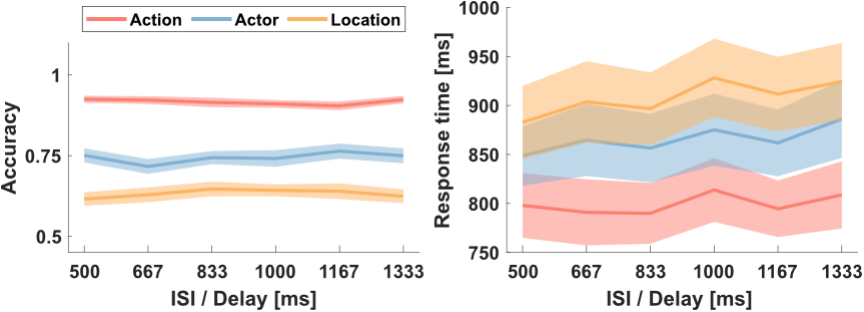
Behavioral performance. Participants performed action matching trials more accurate (left) and faster (right) compared to actor and location trials. Longer ISI slightly increased response times for all trial types. Shaded areas indicate standard errors of the mean.

Participants showed good performance in the task for all ISI and probe types (see Fig. 4A). A 2-way repeated-measures ANOVA for accuracy indicated a main effect of *probe type* (F(2,44) = 159.28, p < .001, ηp^2^ = .835). There was no significant main effect of *ISI* (F(5,110) = 0.28, p = .922, ηp^2^ = .007), and no significant interaction between *probe type* and *ISI* (F(10,220) = 0.88, p = .552, eta^2^ = .039). Regarding the main effect of *probe type*, participants performed significantly better in judging the match between S1 and S2 based on the type of action (91.7 ± 4.1% correct; MEAN ± SD) in comparison to the actor (74.4 ± 8.8% correct) and the location (63.3 ± 7.3% correct), with significant differences, based on post-hoc t-tests, between all condition pairs (all p < .001).

For response times (Fig. 4B), a 2-way repeated-measures ANOVA indicated no significant interaction between *probe type* and *ISI* (F(10,220) = 0.64, p = .778, ηp^2^ = .028), but a significant main effect both for *probe type* (F(2,44) = 24.20, p < .001, ηp^2^ = .682) and *ISI* (F(5,110) = 4.31, p = .001, ηp^2^ = .124). Regarding probe type, we observed the same pattern as for accuracies. Participants were fastest matching actions (799 ± 154 ms), followed by actors (866 ± 170 ms) and locations (908 ± 185 ms), with significant differences, based on post hoc t-tests, between all condition pairs (all p < .05). Response times were slower for longer ISI (1000–1333 ms) compared to shorter ISI (500–833 ms; t(22) = 3.33, p = .003).

### 3.2 EEG-based representational similarity analysis

#### 3.2.1 Standard EEG-RSA

**Fig. 5.**
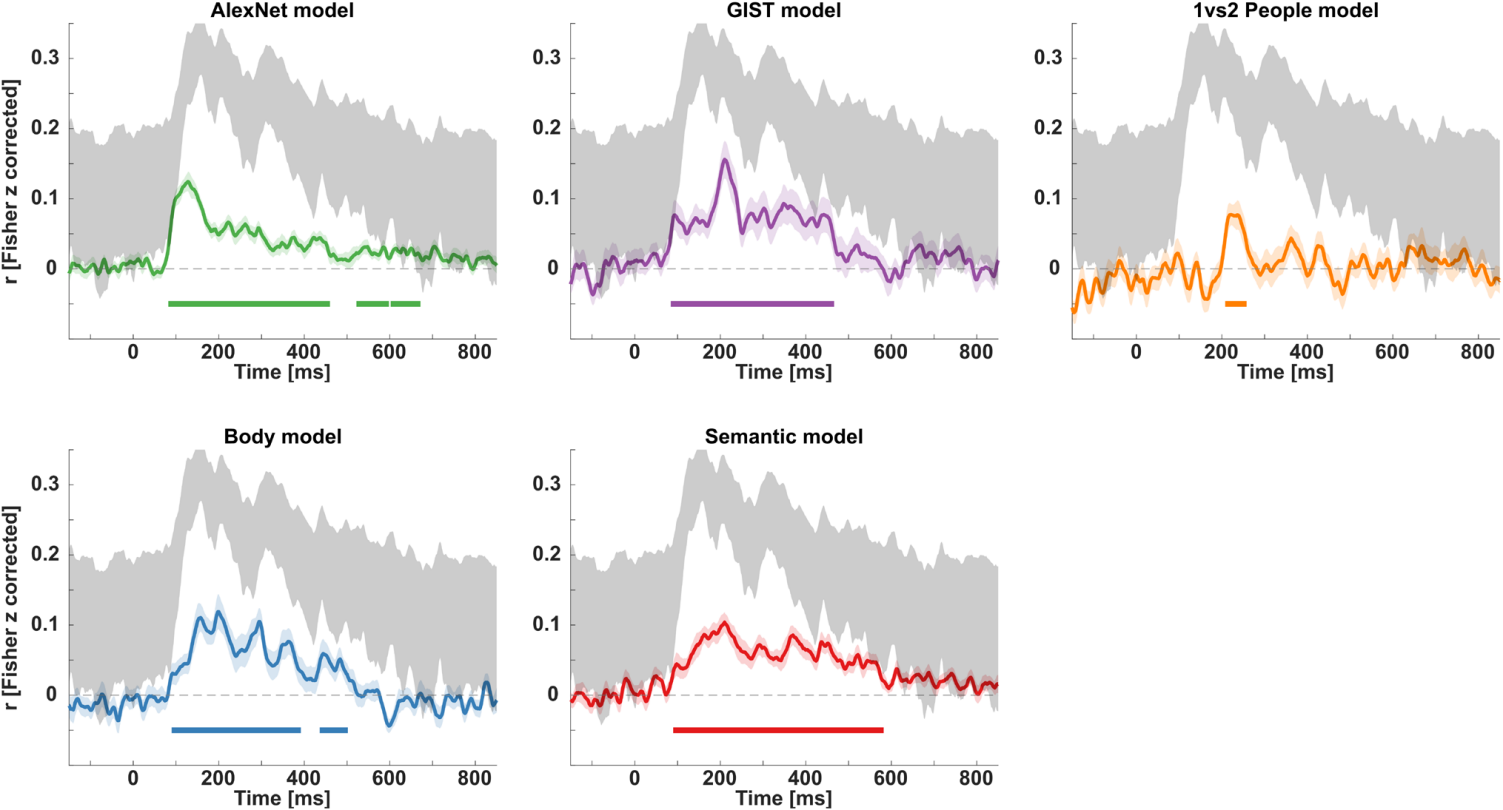
EEG-based standard RSA results. Time courses of all correlations between model RDMs with EEG data (mean ± SE, displayed as shaded areas). Each plot shows group-level Spearman correlation time courses between an independently established model RDM and neural RDMs based on time-resolved EEG-based decoding accuracies with respect to basiclevel actions. Colored horizontal bars indicate time windows where observed correlations deviate from zero, based on one-tailed randomization tests with cluster-correction across time; 10,000 randomizations, p < .05. Noise ceiling is displayed in shaded gray areas (lower and upper bound; Nili et al., 2014).

Using time-resolved RSA, we examined how representations of features that are assumed to contribute to action recognition unfold in time (see Fig. 2 for an illustration of procedures). We correlated EEG-based dissimilarity matrices (based on decoding accuracies) with a set of five models, corresponding to the dissimilarity between basic-level actions in terms of lower-level visual properties (*AlexNet model*), mid-level features (*GIST model, 1 vs 2 people model, Body model*), and higher-level action semantics (*Semantic model*). As shown by a cluster-based group-level standard RSA, all five features are represented in patterns of the EEG signal (Fig. 5). Specifically, the model RDMs for the *AlexNet model*, the *GIST model*, the *Body model*, and the *Semantic model* show sustained clusters of correlation with neural RDMs from approximately 90 ms until 400–600 ms post-stimulus onset. The model RDM for the *1 vs 2 people model* shows a transient cluster of correlation from approximately 200 to 250 ms post-stimulus onset. The *AlexNet model* shows another cluster between approximately 520 and 670 ms (with a short gap <10 ms). All clusters are significant at p < .05. Full cluster statistics are presented in Supplementary Table S2.

#### 3.2.2 Multiple regression EEG-RSA

**Fig. 6.**
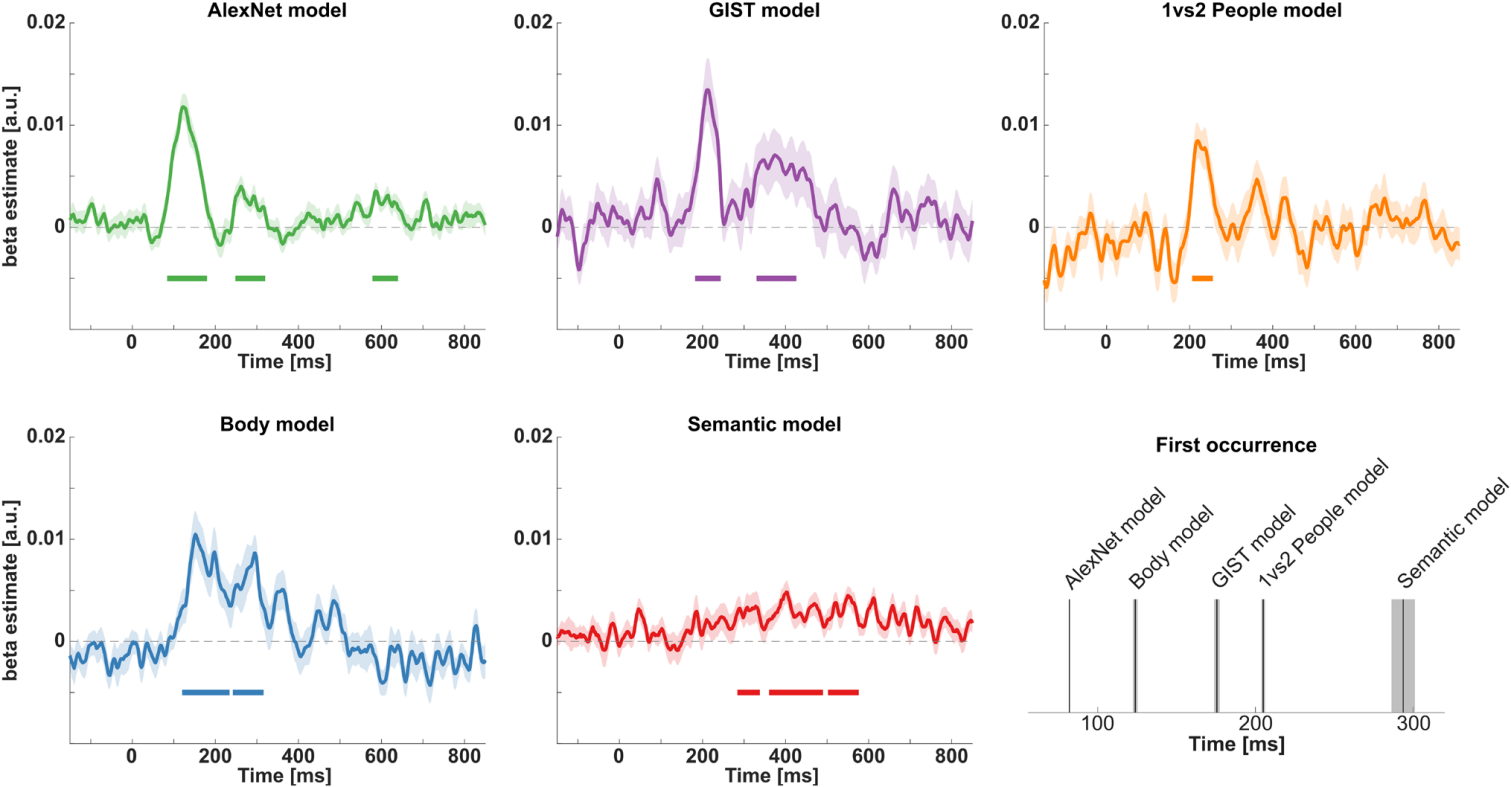
EEG-based multiple regression RSA results. Time courses of multiple regression analyses between model RDMs with EEG data (mean ± SE, displayed as shaded areas). Each plot shows group-level multiple regression parameter time courses between an independently established model RDM and neural RDMs based on time-resolved EEG-based decoding accuracies with respect to actions, accounting for all other model RDMs. Colored horizontal bars indicate time windows where obtained regression parameters are significantly larger than 0, based on one-tailed randomization tests with cluster-correction across time; 10,000 randomizations, p < .05). The lower right plot indicates the time of first occurrence of each model RDM in the EEG data. The shaded area indicates the 95% confidence interval for mean onset latencies, determined by bootstrapping the participant sample.

Following up on the standard RSA, we performed multiple-regression RSA combined with cluster-based randomization analysis to obtain the unique contribution of each model to the resulting brain activity. All models show unique, significant contributions to the EEG patterns recorded during action observation, albeit at different latencies (Fig. 6; see Supplementary Table S3 for full cluster statistics). Specifically, the *AlexNet model* predicted neural RDMs beginning at 82 ms post-stimulus onset (p < .001), followed by the *Body model* at 118 ms (p = .003) and 240 ms (p = .017), the *GIST* and the *1 vs 2 people model* at 180 and 204 ms respectively (p = .008 and p = .019), and finally the *Semantic model* at 282 ms post-stimulus onset (p = .040), as well as 358 ms (p = .002) and 500 ms (p = .007). The *AlexNet model* predicted neural RDMs again after 246 and 576 ms post-stimulus onset (p = .012 and p = .013, respectively), and the *GIST model* again after 328 ms (p = .008), whereas the other models showed single transient (*1 vs 2 people model*, 130 ms) or sustained (*Semantic* and *Body model*, 292 and 196 ms, with short [≤ 22 ms] interruptions between clusters) effects. For the first significant cluster of each model, 95% confidence intervals were determined using bootstrapping (n = 24). Comparing bootstrap estimates for each model, we found that EEG signals were predicted by the *AlexNet model* first (81.6–82.4 ms), followed by the *Body model* (122.3–125.3 ms), the *GIST model* (173.8–177.4 ms), the *1 vs 2 people model* (203.7–206.1 ms), and lastly the *Semantic model* (286.2–301.2 ms). Thus, the 95% confidence interval for each model did not overlap with the confidence interval of the next earlier or later model (Fig. 6).

### 3.3 fMRI-EEG fusion

#### 3.3.1 Whole brain searchlight fMRI-EEG fusion

**Fig. 7.**
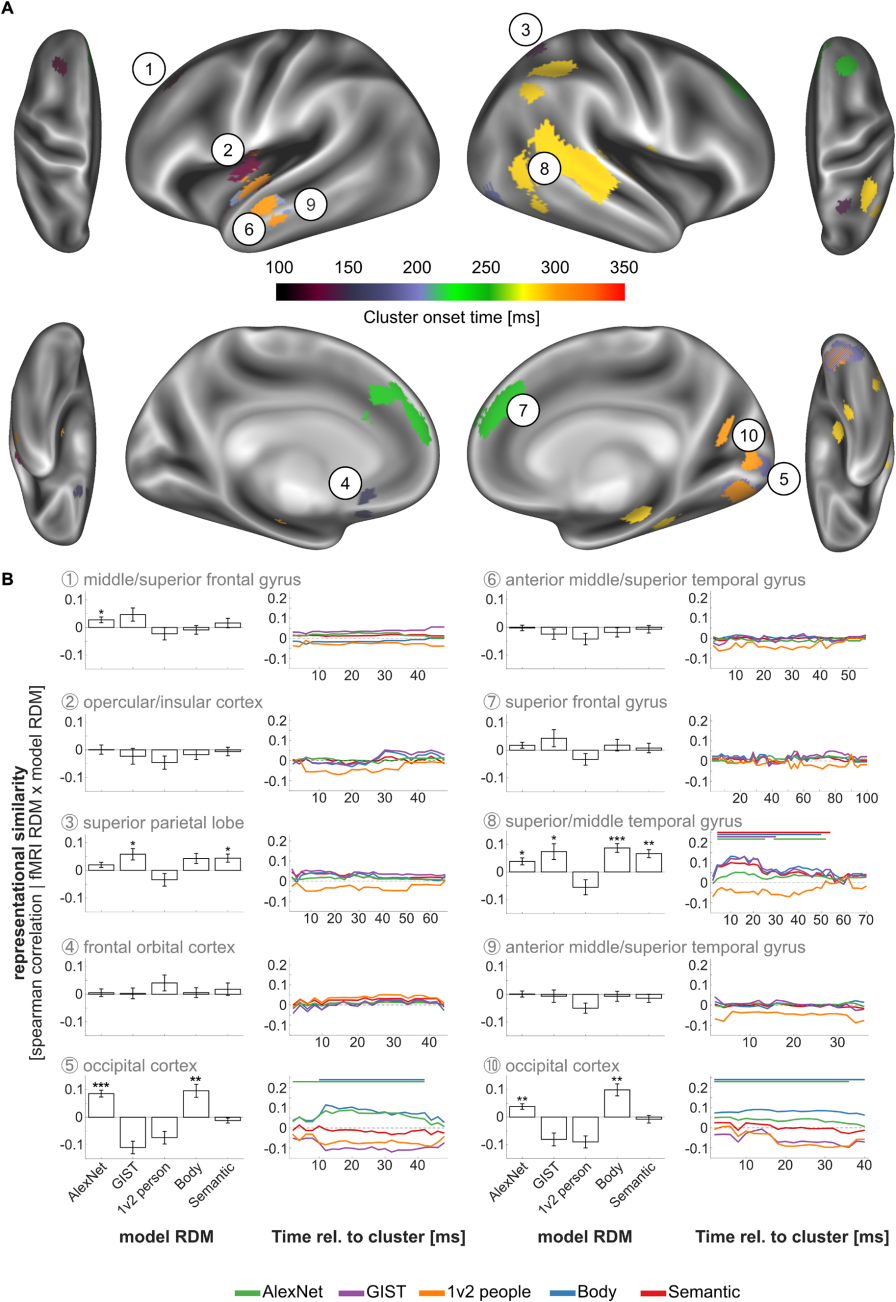
Whole-brain searchlight fMRI-EEG fusion and fusion-based RSA results. (A) Visualization of spatial extent and onset times of fusion-based clusters (purple-yellow-red colour range). Numbers indicate temporal order, and link clusters with corresponding RSA plots (see B). (B) For each cluster (see A), bar plots indicate time-averaged fusion-based RSA results for each of the five models (AlexNet, GIST, 1v2 people, Body, Semantic model), time series plots indicate time-resolved fusion-based RSA results (see Fig. 3 for details). Asterisks denote significant deviations from zero for time-averaged RSA results [***p < .001; **p < .01; *p < .05]; thick horizontal lines indicate extent of significant clusters for time-resolved RSA results. Anatomical cluster labels are based on the Harvard-Oxford cortical structural atlas (see also Table 2 for more details). Error bars indicate the standard error of the mean.

To identify the neural processes underlying action recognition simultaneously in space and time, and subsequently investigating where and when different action features are processed in potentially different brain regions, we carried out whole-brain searchlight fMRI-EEG fusion between the EEG data collected in the current study and the fMRI data obtained by Tucciarelli et al. (2019) using the same stimulus set (see Fig. 3 for an illustration of procedures). This approach exploits the advantages of EEG (high temporal resolution) and fMRI (high spatial resolution) and is based on a representational similarity analysis between data from different modalities, here EEG and fMRI (Cichy & Oliva, 2020). Spatiotemporal cluster randomization tests revealed a significant similarity between the representations captured in the EEG-based and the fMRI-based RDMs based on 10 clusters spanning visual, temporal, parietal, and frontal areas (Fig. 7A; Table 2). The earliest overlap was observed in frontal and insular regions (incl. superior frontal gyrus, middle frontal gyrus (cluster #1), opercular cortex, planum polare (cluster #2), subcallosal cortex, frontal orbital cortex (cluster #4)) and posterior parietal and lateral occipital regions (incl. superior parietal lobe, lateral occipital cortex; cluster #3) around 110 ms, visual areas (incl. lingual gyrus, occipital fusiform gyrus, intracalcarine cortex, occipital pole; cluster #5) around 190 ms, temporal regions (incl. superior and temporal gyrus, planum polare; cluster #6) around 200 ms, frontal regions (incl. superior frontal gyrus, paracingulate gyrus and frontal pole; cluster #7) around 250 ms, temporal regions (incl. middle and superior temporal gyrus, angular gyrus and supramarginal gyrus, cluster #8), around 290 ms, temporal regions (incl. temporal superior and middle temporal gyrus; cluster #9), as well as occipital regions (incl. lingual and occipital fusiform gyrus; cluster #10) around 310 ms (anatomical regions are based on Harvard-Oxford Cortical Structural Atlas in FSL, Desikan et al., 2006; Jenkinson et al., 2012).

**Table 2.** List of fMRI-EEG fusion clusters.

Cluster #	Region	Hem.
1	Middle and superior frontal gyrus	L
2	Opercular cortex, planum polare, insular cortex	L
3	Superior parietal lobe, lateral occipital cortex	R
4	Subcallosal cortex, frontal orbital cortex	L
5	Lingual gyrus, occipital fusiform gyrus, intracalcarine cortex, occipital pole	R
6	Planum polare, middle and superior temporal gyrus	L
7	Superior frontal gyrus, paracingulate gyrus, frontal pole	L/R
8	Superior and middle temporal gyrus, supramarginal gyrus, angular gyrus	R
9	Planum polare, middle and superior temporal gyrus	L
10	Occipital fusiform gyrus, lingual gyrus	R

Anatomical regions are based on the Harvard-Oxford Cortical Structural Atlas. Hem. = Hemisphere, L = left, R = right.

#### 3.3.2 Time-averaged fusion-based RSA

Next, to characterize the nature of the representations captured by the fMRI clusters revealed by the fMRI-EEG fusion analysis (see Section 3.3.1), we identified the representational similarity of these regions with the model RDMs, using temporally unspecific cluster extents (Fig. 7B, bar plots). Five clusters showed significant correlations with model RDMs. The middle/superior frontal cluster (#1) showed a significant positive correlation with the *AlexNet model* (t(19) = 2.72, p(FDR) = .034), but with none of the remaining models (all p(FDR) > .05). The posterior parietal cluster (#3) showed a significant correlation with the *Semantic* (t(19) = 2.99, p(FDR) = .023) and *GIST model* (t(19) = 2.83, p(FDR) = .030), but with none of the remaining models (all p(FDR) > .05). The occipital clusters (#5 and #10) each showed significant positive correlations with the *AlexNet model* (#5: t(19) = 6.94, p(FDR) < .001; #10: t(19) = 3.78, p(FDR) = .005) and the *Body model* (#5: t(19) = 4.03, p(FDR) = .004; #10: t(19) = 4.55, p(FDR) = .001), but not with the remaining models (all p(FDR) > .05). The temporal cluster (#8) showed significant positive correlations with the *Semantic* (t(19) = 4.52, p(FDR) = .001), *Body* (t(19) = 5.57, p < .001), *GIST* (t(19) = 2.60, p(FDR) = .040) and the *AlexNet model* (t(19) = 3.04, p(FDR) = .023), but not with the *1 vs 2 people model* (p(FDR) > .05). The remaining clusters did not show significant correlations with any of the models (all p(FDR) > .05).

#### 3.3.3 Time-resolved fusion-based RSA

Finally, we investigated the temporally resolved representational similarity between fusion-based fMRI clusters and model RDMs (Fig. 7B, time series plots), using sample-specific cluster extents. The occipital clusters #5 and #10 correlated with the *Body model* and the *AlexNet model* for most of the period (all p(FDR) <= .001). The temporal lobe cluster #8 significantly correlated with the *Semantic* (p(FDR) < .001), *Body* (p(FDR) < .001) and *AlexNet model* (p(FDR) = .008 and p(FDR) = .010) RDMs for most of the period, and the *GIST model* during the first half (p(FDR) = .005). The remaining clusters did not show significant correlations with any of the model RDMs (all p(FDR) > .05).

## 4 Discussion

Using fMRI-based multivariate approaches, previous studies have shown that action features at varying hierarchical levels are represented in partially overlapping areas in the LOTC (Kabulska et al., 2024; Tucciarelli et al., 2019; Wurm & Caramazza, 2019; Wurm & Erigüç, 2025; Wurm & Lingnau, 2015; Zhuang et al., 2023). Using EEG-based RSA to examine the time course of action features involved in the recognition of socio-affective actions, action features have been reported to be represented later than visual features, but before social features (Dima et al., 2022). Using fMRI-EEG fusion, and a thorough characterization of the nature of the representations captured by the spatiotemporal clusters obtained in this analysis, the current study supports and extends these findings in an important way, showing that low-, mid-, and high-level features that contribute to the understanding of a wider range of actions (Kabulska & Lingnau, 2023; Lingnau & Downing, 2024, 2015; Wurm & Caramazza, 2022) are extracted in a hierarchical, temporal succession along the lateral pathway.

### 4.1 Unique time courses of neural representations corresponding to specific action features

Using EEG-based RSA, we investigated the temporal evolution of different types of information during action recognition. Using static images of everyday goal-directed actions and models capturing low-, mid-, and high-level features, we revealed the time course of representations corresponding to these features. Using standard RSA, we showed that all five examined features correlated with EEG activity starting 90 ms after stimulus onset (Fig. 5). Features related to lower-level visual properties, scene and body part information, and action semantics showed sustained correlations up to 400–600 ms after stimulus onset. Information regarding the number of people involved in an action showed transient correlations with the EEG signal around 200–250 ms.

More distinguishing findings were obtained using multiple regression RSA, which identified epochs in which each feature uniquely contributed to EEG activity (Fig. 6). Information corresponding to low-level visual features (captured by the *AlexNet model*) emerged at about 80 ms for the first time. Information corresponding to body parts involved in an action (and possibly their movements, as these model RDMs showed high correlations), scene information and the number of involved people, emerged within 100 and 200 ms. Finally, semantic similarities between actions contributed to EEG activity from around 280 ms onward. Interestingly, representations corresponding to visual, body, and scene features re-emerged at later times, suggesting recurrent processing (e.g., Lamme & Roelfsema, 2000; Motlagh et al., 2024; for an extended discussion, see below). Specifically, following an early unique contribution of visual features to EEG patterns between approximately 80–180 ms, visual features again contributed to EEG patterns around 250–300 ms. Similarly, scene information (captured by the *GIST model*) first contributed to EEG patterns around 180–240 ms, and again around 330–400 ms. Visual inspection of Figure 6 suggests clearly separated periods at which these features contribute to neural activity. Moreover, visual inspection of the time course corresponding to the body model also suggests the existence of two separate peaks around 150 and 300 ms, respectively. Visual inspection of the time course corresponding to the semantic model suggests sustained contribution between 280 and 600 ms, despite small gaps between subsequent clusters.

These timing characteristics are in line with previous work showing that visual and action-related features emerge within the first 200 ms (Dima et al., 2022), and that abstract action representations can be observed as early as 200 ms following their presentation (Tucciarelli et al., 2015). Later processing of information regarding the number of actors involved in an action is in line with the same work by Dima et al., who showed activity related to the number of agents emerging around 160 ms. Importantly, whereas Dima et al. applied decoding of neural (EEG) patterns at the level of specific action instances in combination with crowd-sourced ratings regarding visual, action, and social-affective features, we decoded neural patterns generalized to basic level actions—by decoding over different instances of the same action performed by different actors, using different objects, scenes, and kinematics. This allows us to generalize our results to basic-level actions, that is, the level at which category information is assumed to be maximized (Rosch et al., 1976; Zhuang & Lingnau, 2022; Zhuang et al., 2023).

### 4.2 Body, scene, and social information represented before action semantics

Our results suggest a clear temporal order in which action features are represented in the brain. Following lower-level visual features, the brain first represents bodily features of actions, followed by scene information and social properties of the action (i.e., the number of agents involved in the action). Semantic information is represented only at a later stage. This order suggests that properties of actions, such as the effectors and kinematics with which they are performed, and the scene in which they are performed, contribute to the recognition of actions at the level of action goals, corresponding to the representation of action semantics. As such, the outcomes of this study are in line with recent behavioral observations suggesting that the recognition of actions at the level of body postures—corresponding to our level of effects and kinematics—requires shorter exposure durations than the recognition of action-related objects and scenes, and that these features may be processed in parallel, whereas the recognition of action goals requires longer exposure durations and possibly depends on the integration of these features (Reger et al., 2025). Taken together, these results add to recent observations and frameworks suggesting that action recognition relies on a visual analysis of the observed action, including the integration of contextual information, such as object and scene information along the lateral visual pathway (El-Sourani et al., 2018; Lingnau & Downing, 2024; Wurm & Caramazza, 2022; Wurm & Schubotz, 2017). Specifically, midlevel features such as kinematics, object, and scene information may be processed in parallel, before being integrated at the level of action semantics. The observation of a temporal ordering also adds to seemingly conflicting findings in previous studies that reported different feature spaces within the same brain areas, in particular, LOTC (Kabulska et al., 2024; Tarhan & Konkle, 2020; Tucciarelli et al., 2019; Zhuang et al., 2023; see also Lingnau & Downing, 2024).

### 4.3 Feedback, re-emerging representations

Using multiple regression RSA, we not only showed a temporal order in which action features are represented in the brain, but also observed that representations corresponding to visual, scene, and body features re-emerge following an initial early representation (Fig. 6). Specifically, the second period at which visual features uniquely contribute to EEG patterns falls after the first representation of body and scene information (2^nd^ cluster *AlexNet model*, 250-300 ms); the second epoch at which body features contribute to EEG patterns falls after the first epoch of scene information, and at the same time of the second representation of visual features (2^nd^ peak *Body model*, 250–300 ms); and the second period at which scene features contribute to EEG patterns falls after the second representation of body features (2^nd^ cluster *GIST model*, 350–400 ms).

Pure feedforward processing, both serial and parallel (extracting different action features in parallel), would not require re-emerging neural representations; hence these observations suggest that action recognition also involves recurrent processing. The pattern of results suggests that, in particular, processing of body and scene information takes places in feedback loops with processing of lower-level visual features, and possibly, extraction of semantic information takes place in feedback loops with processing of scene and body information. As such, in contrast to accounts relying on feedforward processing of movement-related information (Gallese et al., 1996), the possible existence of feedback loops during action recognition is in line with predictive coding accounts of action understanding (c.f., Cerliani et al., 2022; Kilner, 2011; for a recent discussion, see also Lingnau & Downing, 2024).

Related to the previous point, it can be assumed that the restriction to static pictures as stimuli in the current study, with no prior information regarding any of the action features, contrasts with naturalistic action perception. In fact, during naturalistic action perception, some information, such as the scene where an action takes place, or a selection of objects, can usually be perceived and recognized before an action unfolds. Accordingly, additional predictive processes might take place, that predict higher-level abstract action features before lower-level concrete features (see de Vries & Wurm, 2023, for a demonstration of a cascade of predictive neural representations of observed actions from abstract to concrete features). However, we would expect the same processing of features and possibly, their interaction, during the recognition of dynamic actions as we have observed here during the recognition of static actions, in line with studies showing that action categories can be decoded across visual formats in inferior parietal, occipitotemporal, premotor and middle frontal cortex (Hafri et al., 2017).

### 4.4 Spatiotemporal dynamics of neural representations underlying action recognition

A whole-brain searchlight fMRI-EEG fusion analysis revealed representational overlap during early time windows in both frontal and parietal regions, before early and higher visual areas, and lastly occipito-temporal regions (Fig. 7A; Section 3.3.1). To characterize the functions of these regions with respect to action recognition, we related fMRI-based RDMs from each region with the five different model RDMs. This analysis (Fig. 7B; Sections 3.3.2 and 3.3.3) revealed that early activated parietal and frontal regions are likely not related to representations of action-related features, as indicated by a lack of correlation with any of our feature models (however, it should be noted that a cluster spanning superior parietal and lateral occipital regions correlated with the semantic feature model and scene model in a time-unspecific, but not a time-specific RSA, and a cluster in the middle/superior frontal gyrus correlated with the AlexNet model in a time-unspecific, but not a time-specific RSA; see Sections 3.3.2 and 3.3.3 for details). The analyses further revealed that occipital regions represent information captured in the *Body model* and the *AlexNet model*. These regions, therefore, likely code lower-level visual information and body- or movement-related properties. Finally, occipito-temporal regions contain information captured in the *AlexNet*, *GIST*, *Body*, and *Semantic model*. Importantly, the LOTC is shown to represent, partially in temporal order, visual, scene, body, and semantic information, as revealed by the time-resolved fusion-based RSA (see Section 3.3.3). These findings are in line with the idea that the LOTC represents and integrates action features across different levels of specificity, and crucially, suggest that the representations in ventral/lateral stream areas are sufficient to recognize observed actions (Dima et al., 2022; Lee Masson & Isik, 2023; Lee Masson et al., 2024; Lingnau & Downing, 2024; Wurm & Caramazza, 2019; Wurm & Lingnau, 2015).

Several identified clusters did not show any correlation with the model RDMs, particularly not during a time-resolved analysis. The role of these, in particular frontal, regions may be related to the task or general attentional processes that take place in both experiments during which the data were collected, that is, the delayed matching task that was performed in the present study, and the 1-back task performed in the study by Tucciarelli et al (2019). Regions in the inferior and superior parietal cortex have been shown to be sensitive to the difference between actions at the basic and superordinate level (Abdollahi et al., 2013; Hafri et al., 2017; Jastorff et al., 2010; Urgen & Orban, 2021; Zhuang et al., 2023). While our results are not incompatible with these observations, the temporal dynamics of the representation of action-related features revealed in the current study are in line with the view that the representations obtained in parietal regions are of secondary nature to action recognition and may serve other purposes, for example, the preparation of potential motor responses (see e.g., Lingnau & Downing, 2024 for a discussion). Together, our findings extend previous ROI-based work by Lee Masson et al. (2021, 2023, 2024), showing that the posterior superior temporal sulcus (STS) and MTG, but not frontoparietal regions, represent social features of actions during viewing of social actions in artificial or movie scenes.

### 4.5 Limitations and future directions

As with most studies investigating representations of goal-directed, everyday actions, the current work is restricted to 27 actions, spanning five larger action categories (locomotion, social/communicative, cleaning-related, food-related, leisure-related; see Tucciarelli (2019) for details). Other studies have started to vastly extend this space (e.g., Bockes, Hebart, Lingnau, subm.; Dima et al., 2023; Kabulska & Lingnau, 2023; Kabulska et al., 2024; Thornton & Tamir, 2022; Vinton et al., 2024). Here, we decided to use a limited number of actions for two reasons. First, we used relatively controlled stimuli, with two fixed actors and two locations per action, controlled viewpoints and distances; studies that use larger action spaces are often based on less controlled stimuli (e.g., large collections of images available in online databases), and/or real or scrambled movies, which have less clear onset times (Dima et al., 2022; Lee Masson et al., 2024). Here, we focus on more repetitions of the same action using controlled properties while extracting information that is general to an action at the basic level, in particular by collapsing over different instances of the same basic-level action performed by various actors, in different locations, and using a variety of tools and objects. Second, we used a stimulus set that already includes fMRI data from an independent sample of 20 participants (Tucciarelli et al., 2019), allowing us not only to use behavioral ratings and models from this work, but also to combine the fMRI data with our EEG data in an EEG-fMRI fusion approach, thereby extending the utility of the dataset.

We would like to point out that the stimulus set used in the current study was not fully balanced with respect to all contextual and body features, which is partly due to naturalistic statistics (e.g., some actions are more likely to take place indoors or involve particular objects and kinematics than others). Consequently, our model RDMs were not independent of each other, and some models that we initially planned to use had to be excluded to allow us to perform multiple regression analyses (see Section 2.1.6 for details). This became particularly problematic for the *Body* and *Movement models*, as well as for the *Semantic*, *Object*, and *Context model*. We, therefore, reduced the model set by dropping the movement model, and by dropping both the *Object* and *Context* (‘where an action usually takes place’) *model*, and introduced a computational model (Oliva & Torralba, 2001) to capture scene information contained in the action images, in the *GIST model*. Therefore, the current data do not allow to fully disentangle different properties related to the actual movements of an agent (i.e., *which part* of the body is moved, and *how* is it moved), and semantic representations of actions might be affected by similarities with respect to the objects used in an action. These problems will need to be solved in future studies, potentially by using a stimulus set that is balanced with respect to contextual and body features, and/or that includes movement information obtained from kinematic recordings or computational modelling.

Whereas we are confident that our fMRI-EEG fusion analysis provided valuable data, the results need to be interpreted carefully. Most notably in the light of our interpretation of the findings, the cluster analysis revealed lateralization effects within the lateral visual pathways (i.e., cluster 6, 8, and 9; Fig. 7). Whereas most studies report action-related effects in bilateral LOTC (e.g., Hafri et al., 2017; Kabulska et al., 2024; Wurm & Lingnau, 2015; Wurm et al., 2016; Zhuang et al., 2023) or left LOTC (Tucciarelli et al., 2019), some effects appear stronger in the right hemisphere (e.g., Wurm et al., 2017), and the causes for these lateralization effects are not fully understood. In fact, an inspection of sub-threshold clusters (p < .10) indicated smaller fusion-based clusters of shorter duration, at comparable latencies, within the left LOTC. Given that the current study was not optimized for a fusion analysis, future studies are required to generate optimized data, especially by combining EEG and fMRI data from the same participants.

Finally, the task in this study was designed to take away the focus of specific action features, by withholding the probe for the matching task until after the second stimulus has been perceived. Hypothetically, if participants were informed about the focus of a trial (i.e., which feature to use for matching two actions) prior to presenting the actions, the sensitivity of different brain regions would likely have changed. In particular, we would expect the processing of action information to be more emphasized within the fronto-parietal cortex, where it has been shown that explicit processing of actions, in contrast to implicit processing, increases decoding accuracy, especially for concrete, but not abstract, levels of actions—whereas LOTC showed decoding irrespectively of task and action level (Wurm et al., 2016).

## 5 Conclusion

Using EEG-based RSA during action recognition, we characterized temporal dynamics of neural representations underlying the extraction of semantic action information. Our results show that different action features are represented in a temporal order—from visual information to body and scene information to semantic information, and likely involve feedback loops between these stages. The results of our fMRI-EEG fusion analysis further suggest that LOTC plays a central role representing action features at different hierarchical levels, as well as their integration toward semantic representations. As such, our results are in line with recent theories suggesting that action recognition entails the integration of concrete contextual properties in occipital and temporal brain regions. These findings support ideas suggesting that the lateral visual pathway integrates information from a variety of sources, such as movement kinematics, objects, and scenes, to form an integrated representation of observed actions (c.f., Jung et al., 2025; Lingnau & Downing, 2024; McMahon & Isik, 2024; Urgen & Orban, 2021). It will be an important goal for future studies to further characterize both the spatiotemporal dynamics, the exact features that are required to recognize actions, and the causal contributions of each of those features to the process of action recognition.

## Supplementary Material

Supplementary Material

## Data Availability

Preprocessed data and analysis code are available via the OSF repository: https://osf.io/qbjry/.
