# Supplementary material for: The spatiotemporal neural dynamics of action-related features underlying action recognition
Source: Imaging Neurosci (Camb). 2025 Nov 13;3:IMAG.a.1019. doi: 10.1162/IMAG.a.1019 (PMC12616154; doi:10.1162/IMAG.a.1019)
Supplement: Supplementary Material [file IMAG.a.1019_supp.pdf]

Supplementary Materials

The spatiotemporal neural dynamics of action-related features underlying action recognition

Marius Zimmermann[1], Angelika Lingnau[1]

[1] Chair of Cognitive Neuroscience, Institute of Psychology; University of Regensburg, 93053 Regensburg, Germany

Corresponding author: marius.zimmermann@ur.de

S1: Pearson correlation coefficients between model RDMs for all models

	Semantic	Body	Context	Move-ment	Object	AlexNet	GIST	1vs2 People
Semantic	1	0.47	0.83	0.64	0.74	0.21	0.19	0.15
Body		1	0.44	0.74	0.44	0.16	0.34	-0.01
Context			1	0.66	0.69	0.28	0.32	0.05
Movement				1	0.61	0.2	0.31	-0.01
Object					1	0.12	0.06	0.05
AlexNet						1	0.36	0.14
GIST							1	-0.05
1vs2 People								1

S2: Cluster based randomization statistics, EEG-based standard RSA

Alexnet model:

Cluster #1	80 - 458 ms	p = 0.000	***
Cluster #2	600 - 670 ms	p = 0.017	*
Cluster #3	520 - 596 ms	p = 0.020	*
Cluster #4	694 - 718 ms	p = 0.133	
Cluster #5	744 - 754 ms	p = 0.399	
Cluster #6	682 - 686 ms	p = 0.563	
Cluster #7	484 - 488 ms	p = 0.583	
Cluster #8	466 - 468 ms	p = 0.621	
Cluster #9	764 - 764 ms	p = 0.649	
Cluster #10	780 - 780 ms	p = 0.662	

GIST model:

Cluster #1	82 - 464 ms	p = 0.000	***
Cluster #2	666 - 666 ms	p = 0.693	

1vs2 People model:

Cluster #1	206 - 256 ms	p = 0.017	*
Cluster #2	356 - 370 ms	p = 0.420	

Cluster #3	636 - 640 ms	p = 0.647	

Body model:

Cluster #1	88 - 390 ms	p = 0.000	***
Cluster #2	434 - 500 ms	p = 0.027	

Semantic model:

Cluster #1	88 - 580 ms	p = 0.000	***
Cluster #2	644 - 694 ms	p = 0.058	
Cluster #3	712 - 750 ms	p = 0.090	
Cluster #4	604 - 622 ms	p = 0.227	
Cluster #5	44 - 62 ms	p = 0.282	
Cluster #6	626 - 638 ms	p = 0.373	
Cluster #7	780 - 788 ms	p = 0.439	

S3: Cluster based randomization statistics, EEG-based multiple regression RSA

AlexNet model:

Cluster #1	82 - 178 ms	p = 0.000	***
Cluster #2	246 - 318 ms	p = 0.012	*
Cluster #3	576 - 638 ms	p = 0.012	*
Cluster #4	532 - 554 ms	p = 0.222	
Cluster #5	700 - 716 ms	p = 0.275	
Cluster #6	778 - 778 ms	p = 0.800	

GIST model:

Cluster #1	328 - 424 ms	p = 0.008	*
Cluster #2	180 - 242 ms	p = 0.009	*
Cluster #3	428 - 450 ms	p = 0.289	
Cluster #4	86 - 96 ms	p = 0.514	

1vs2 People model:

Cluster #1	204 - 254 ms	p = 0.018	*
Cluster #2	354 - 370 ms	p = 0.389	

Body model:

Cluster #1	118 - 232 ms	p = 0.003	**

Cluster #2	240 - 314 ms	p = 0.017	*
Cluster #3	342 - 374 ms	p = 0.139	
Cluster #4	478 - 498 ms	p = 0.276	
Cluster #5	448 - 452 ms	p = 0.668	

Semantic model:

Cluster #1	358 - 488 ms	p = 0.002	**
Cluster #2	500 - 574 ms	p = 0.007	*
Cluster #3	282 - 336 ms	p = 0.040	*
Cluster #4	588 - 622 ms	p = 0.054	
Cluster #5	38 - 58 ms	p = 0.207	
Cluster #6	650 - 668 ms	p = 0.261	
Cluster #7	708 - 724 ms	p = 0.272	
Cluster #8	778 - 790 ms	p = 0.404	
Cluster #9	682 - 692 ms	p = 0.454	
Cluster #10	740 - 748 ms	p = 0.471	
Cluster #11	244 - 248 ms	p = 0.621	
Cluster #12	184 - 186 ms	p = 0.644	
